# When Digital Twin Meets Network Softwarization in the Industrial IoT: Real-Time Requirements Case Study

**DOI:** 10.3390/s21248194

**Published:** 2021-12-08

**Authors:** Mehdi Kherbache, Moufida Maimour, Eric Rondeau

**Affiliations:** CRAN Laboratory, Université de Lorraine, CNRS, UMR 7039, Campus Sciences, BP 70239, F-54000 Nancy, France; moufida.maimour@univ-lorraine.fr (M.M.); eric.rondeau@univ-lorraine.fr (E.R.)

**Keywords:** Digital Twining, Internet of Things, industry 4.0, Software Defined Networks

## Abstract

The Industrial Internet of Things (IIoT) is known to be a complex system because of its severe constraints as it controls critical applications. It is difficult to manage such networks and keep control of all the variables impacting their operation during their whole lifecycle. Meanwhile, Digital Twinning technology has been increasingly used to optimize the performances of industrial systems and has been ranked as one of the top ten most promising technological trends in the next decade. Many Digital Twins of industrial systems exist nowadays but only few are destined to networks. In this paper, we propose a holistic digital twinning architecture for the IIoT where the network is integrated along with the other industrial components of the system. To do so, the concept of Network Digital Twin is introduced. The main motivation is to permit a closed-loop network management across the whole network lifecycle, from the design to the service phase. Our architecture leverages the Software Defined Networking (SDN) paradigm as an expression of network softwarization. Mainly, the SDN controller allows for setting up the connection between each Digital Twin of the industrial system and its physical counterpart. We validate the feasibility of the proposed architecture in the process of choosing the most suitable communication mechanism that satisfies the real-time requirements of a Flexible Production System.

## 1. Introduction

The Internet of Things (IoT) is experiencing strong growth in various fields and is constantly evolving, with a forecast of more than 125 billion connected objects in the world by 2030 [1]. These are mainly wireless sensors connected to the Internet but also any other physical or virtual object that can communicate via this global network. The IoT is foreseen to play a vital role in the fourth industrial revolution with the advent of Industrial IoT (IIoT) paving the way for a wide range of industrial applications to benefit from full automation and high productivity. Powerful industrial systems can be designed through the deployment of wireless sensors, actuators, controllers and other smart devices.

Facilitated by this dramatic development of the IIoT along with the rise of big data analytics, the last decade witnessed the revival of the concept of Digital Twining (DT) [2]. In particular, the IoT allows for keeping a digital twin consistent and synchronized with the physical entity it represents thanks to its sensing technology coupled with the communication capabilities it provides. The digital and physical twins in addition to the IoT ensuring the twins connection form a Cyber-Physical System (CPS). Digital Twining is ranked by Gartner [3] as one of the top ten most promising technological trends for the next decade. Digital Twining is particularly promising in creating a continuously updated model of a physical system to enable rapid adaptation to dynamics mainly unpredicted and undesirable changes. A wide range of industrial fields are concerned [4] such as manufacturing [5,6,7], healthcare [8,9], maritime and Shipping [10,11], city management [12,13], and aerospace [14,15].

In Cyber-Physical Production Systems (CPPS) and industry 4.0, digital twins are made for physical assets that compose the industrial system. The major effort is underway to narrow any gaps that may occur between the twins. Some CPPS architectures are proposed [16,17], however, the communication network connecting the twins was omitted despite its vital importance in the whole CPPS. Recently, some DT architectures for networks have been proposed. The architecture proposed in [18] only aims to provide adaptive routing in software defined vehicular networks. While the Internet Engineering Task Force (IETF) (IETF is a large open international community of network designers, operators, vendors, and researchers concerned with the evolution of the Internet architecture and the smooth operation of the Internet) draft [19] presents a reference architecture of a Digital Twin Network, the work is not yet mature and it does not target the Industrial IoT with a holistic approach. Once again, ref. [20] discusses the opportunities that could provide Digital Twinning to fulfill the potentials of 5G networks without considering an industrial system.

In this paper we propose a holistic Network Digital Twin (NDT) architecture for the IIoT to enable closed-loop network management across the entire network life-cycle. This allows for movement from the current network design methodology to a more dynamic one. In fact, the NDT allows to leverage the output from both twins to suggest improvements on the designed networking protocols and algorithms. An ongoing evolution of the physical network is possible by taking actions during the network service phase with the objective of maximizing the performance. In practice, we chose to leverage the Software Defined Networking (SDN) paradigm as an expression of network softwarization. SDN decouples the data plane from the control plane and allows centralized network orchestration [21]. Controllers form the control plane and hold the control of the network by sending instructions and commands to the devices in the data plane. They also collect the required information from the physical network to build a centralized global view. This two-way connection can be exploited by the NDT for real-time network monitoring, predictive maintenance mechanisms, and network diagnostics.

To validate our proposed architecture, an industrial project aiming to connect a Flexible Production System (FPS) to the Internet using sensor networks is considered. The concept of NDT is used in the early stage of the project. One design issue consists in choosing the communication mechanism that suits the real-time requirements of the FPS application. An NDT is built to allow the assessment of different networking policies that aim to achieve reliability and timeliness prior to the deployment of the most suitable one. To the best of our knowledge this is the first work that introduces the concept of network digital twin for the IIoT based on a holistic approach. The remainder of the paper is organized as follows. Section 2 presents some important concepts used to design our architecture along with some related work. Section 3 describes our proposed architecture while Section 4 details the use case we considered. Section 5 concludes the paper and discusses some future work.

## 2. Background and Related Work

In this section, we present some notions that make the fundamental bricks of our architecture. First of all, we provide a brief definition of the SDN paradigm along with its architecture. Then we provide an overview of the Digital Twinning concept covering its origins and its main notions. Finally, we discuss some existing network specific DT architectures as related works and the main characteristics that make our architecture original.

### 2.1. Software-Defined Networks (SDN)

**Figure 1:**
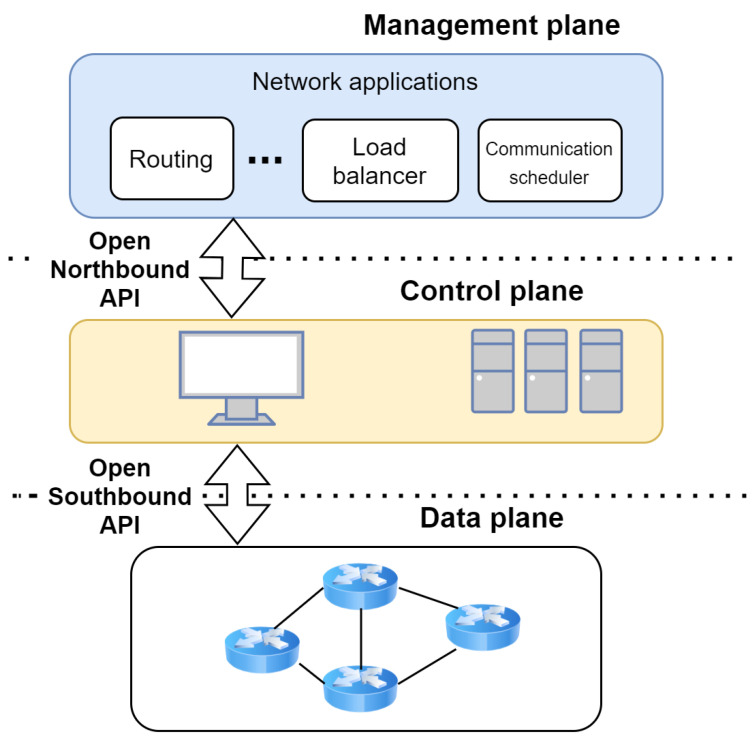
Simplified view of SDN architecture.

According to the Software-Defined Networking paradigm, computer networks can be split in three planes: management, control and data planes as depicted in Figure 1. The data plane is composed of the forwarding devices (switches, routers, etc.). The control plane is responsible for sending commands to the forwarding devices in order for them to apply the required networking policy. The management plane is represented by network management applications such as those related to traffic engineering, mobility management and wireless communications, security, and reliability. These applications are implemented using network programming languages and they interact with the control plane thanks to an open northbound Application Programming Interface (API). The control plane controls the forwarding devices via an open southbound API such as OpenFlow [22].

This three-plane division allows for more flexibility, scalability, and performance compared to traditional networking. In fact, SDN abstracts network intelligence from forwarding devices to a logically centralized controller. This simplifies network management, configuration, and evolution [23]. It can be defined as a network architecture with four characteristics according to Kreutz et al. [21]: (i) the control and data plane are decoupled; (ii) flow-based forwarding, meaning that routing decisions will be made based on packet properties instead of its destination, which offers high network flexibility; (iii) control logic is moved to an external entity, called SDN controller, which facilitates the programming of network devices by providing an abstract global view of the network; (iv) the network is programmable by the means of software applications implemented on top of the control logic.

### 2.2. Digital Twining

A *Digital Twin* (DT) can be viewed as a machine that is emulating or “twinning” the life of a physical entity [24]. A DT is more than just a simple simulation or a static model, it is a continuously evolving model that is always aware of the events happening in its physical twin as it follows its lifecycle to supervise and optimize its functions. The synchronization between the DT and its physical counterpart is possible thanks to the real-time data uploading ensured by IoT devices and sensor technology, while big data storage capabilities allow keeping historical data that can be useful for the Digital Twin. AI algorithms, for instance, can be used to predict future states of the physical twin. A DT can also simulate new configurations in order to apply preventive maintenance operations [24]. Theses properties result in reducing costs and resources in many industries such as manufacturing and healthcare systems.

DT as a concept includes a real space, a virtual space and information/data exchange between the two spaces. It was first introduced in 2002 by Professor Grieves in the Product Lifecycle Management (PLM) course of the University of Michigan [25]. The concept went through a slow-going development phase from 2003 to 2011 where only few articles were published [26]. Right after 2011, the availability of low-cost sensors and communication technologies in the era of the IoT, the emergence of big data analytics and simulations technologies, the remarkable advance in Machine Learning (ML) along with powerful computation infrastructure, triggered the rise of DT technology. It gained widespread popularity among researchers in both academia and industry when NASA gave a formalized definition of DT and how it would ameliorate performance in the astronautics and aerospace field [27]. In 2014, Grieves published the first white paper extending the DT concept from one conceptual idea to various practical applications [28]. Since then, numerous DT applications have appeared and DT technology has been classified as one of the top ten most promising technology trends in the next decade by Gartner in 2017 and 2018 [3]. More recently, control-oriented DTs are emerging. In [29] is proposed a digital twin offering uncertainty management and robust process control.

### 2.3. Digital Twin Architectures

Some network specific DT architectures have been proposed in the literature. A Digital Twin enhanced Industrial Internet (DT-II) reference framework towards smart manufacturing is proposed in [30]. The interplay between digital twin and Industrial Internet is discussed in three aspects of product, enterprise, and business. On the one hand, they show how the sensing/transmission network capability of Industrial Internet can provide data acquisition and transmission to digital twins. On the other hand, they show how the physical-virtual real-time synchronization feature of digital twin is consistent with the interconnection of intelligent machines required by Industrial Internet. The Intelligent Digital Twin-based Software-Defined Vehicular network (SDVN) [18] has been designed to allow intelligent and adaptive routing in vehicular networks. By having a DT that replicates the vehicular network in real-time, a report is sent to it when a routing error occurs. The DT verifies different routing schemes before choosing the one with the best performance to deploy in the physical network.

The IETF group is currently working on standardizing the concept of Digital Twin Network (DTN). The last draft [19] presents the basic concepts and a reference architecture along with the main challenges for building a DTN. The presented DTN in this work is for computer networks in general and does not consider the specificity of the IIoT. A 5G Digital Twin is presented in [20] to help developing and deploying 5G complex networks and permit cost-effective access to 5G knowing that deploying 5G networks is too expensive. In [31], an Application-driven Digital Twin Networking (ADTN) middleware is proposed in order to simplify the interaction with heterogeneous distributed industrial devices and dynamic management of network resources by adopting an application-level point of view. This architecture adopts an SDN-based cross-layer approach to dynamically orchestrate the industrial environment while taking into consideration Quality of Service (QoS) requirements and network configuration adaptation capabilities. The leading communications service provider, Huawei, proposed Intent-Driven Network (IDN) [32] which builds a digital twin between the physical network and business intent with the goal of helping enterprises accelerate business innovation and boost operation efficiency. The built digital twin could enable quick detection and resolution of network problems, prediction of future network status and enhancement of network reliability by eliminating network risks in advance.

Our architecture is targeted to IIoT networks, uses SDN as the communication interface between the digital and the physical world and considers the interaction between the Network Digital Twin and other Digital Twins in an industrial environment.

## 3. A Holistic Digital Twinning Architecture for the IIoT

The process of designing and validating network solutions can go through a theoretical analysis as a preliminary step to prove the underlying algorithms convergence and their correctness. On the other hand, simulation (or emulation) tools are widely used by network researchers to develop and evaluate their algorithms and protocols. This is due to the fact that these tools are a good way to quickly test protocols on a large scale at a low cost. To evolve the developed solution, the process is repeated as in Agile methodology using inputs from the previous steps and eventually from experimental validation and deployment steps, until it is fully functional and ready for deployment. These iterations are done off-line implying human intervention which makes this approach prone to errors. We argue that the problem of this methodology is the lack of connection with the real world network throughout the entire life cycle from the first to the final phase. In other words, the coordination between the different steps can be quite challenging. Moreover, with this approach, the designed solution can only be completely validated at the end of the deployment phase. This is further exacerbated in the context of Wireless Sensor Networks (WSN), a basic building block of the IoT.

We estimate that the industrial IoT is a complex system since it is characterized by a large network of components, many-to-many communication channels and sophisticated information processing that makes prediction of system states difficult [33]. We would contend that complex systems have a major element of surprise, as in “I didn’t see that coming”. That surprise is generally, although not always, an unwelcome one. So the element of surprise is not to be ignored. In fact, in the real world many factors can impact the operation of a WSN: outdour conditions, weather conditions, radio interference from other wireless technologies, etc. The LOFAR-agro project presented in [34] is the perfect example on how things can go extremely wrong after deployment. The project members intended to deploy a large scale WSN of up to 100 nodes for a pilot in precision agriculture. Everything worked fine in simulation and in short-scale deployment (10 nodes), but when coming to the real world deployment, they faced an endless stream of hardware malfunctions, programming bugs, software incompatibilities, combined with the harsh nature conditions and time pressure. That made them face unsolvable problems due to the layering of the different problems and, in our opinion, the lack of continuous connection between the real world network and the design/validation process.

In the networking field, it is known that simulation is not a tool for fully validating a solution since it cannot take into account all the environmental variables surrounding the network. Although, it helps gaining a better understanding of current performances. On the other hand, deployment testing is costly in terms of time and money. That is why, we are proposing a novel Digital Twinning based architecture for the IIoT that should permit closed-loop network management across the entire network life-cycle from the early design stages to the service and maintenance phases. To do so, we suggest to introduce the concept of the Network Digital Twin (NDT).

**Figure 2:**
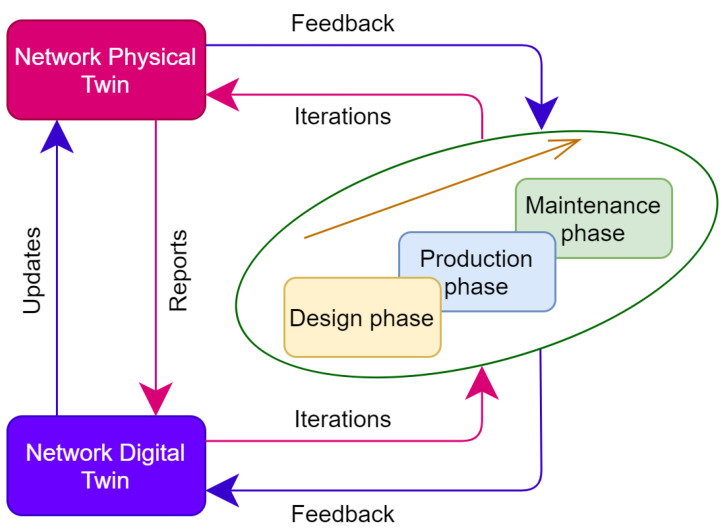
NDT role throughout the whole network development process.

By creating a digital twin of the industrial network, a *’living model’* that is kept constantly updated, decisions are, therefore, made based on current conditions rather than those of the original study. As depicted in Figure 2, modeling and analysis can be tightly coupled with execution, enabling a cycle of continuous improvement and innovation. Enhancing network reliability and dealing with network risks in advance by predicting future network status using, for instance, AI algorithms in the digital twin. Improved performance can also be achieved by adjusting network configuration based on different options, adaptation to evolving traffic and resource demands and by experimenting safely different solutions to determine the optimal configuration of networks without jeopardizing the operation of the physical network. This eliminates the risks related to testing new network policies in a production environment and decreases the corresponding costs since the experiments are done in the network digital twin.

**Figure 3:**
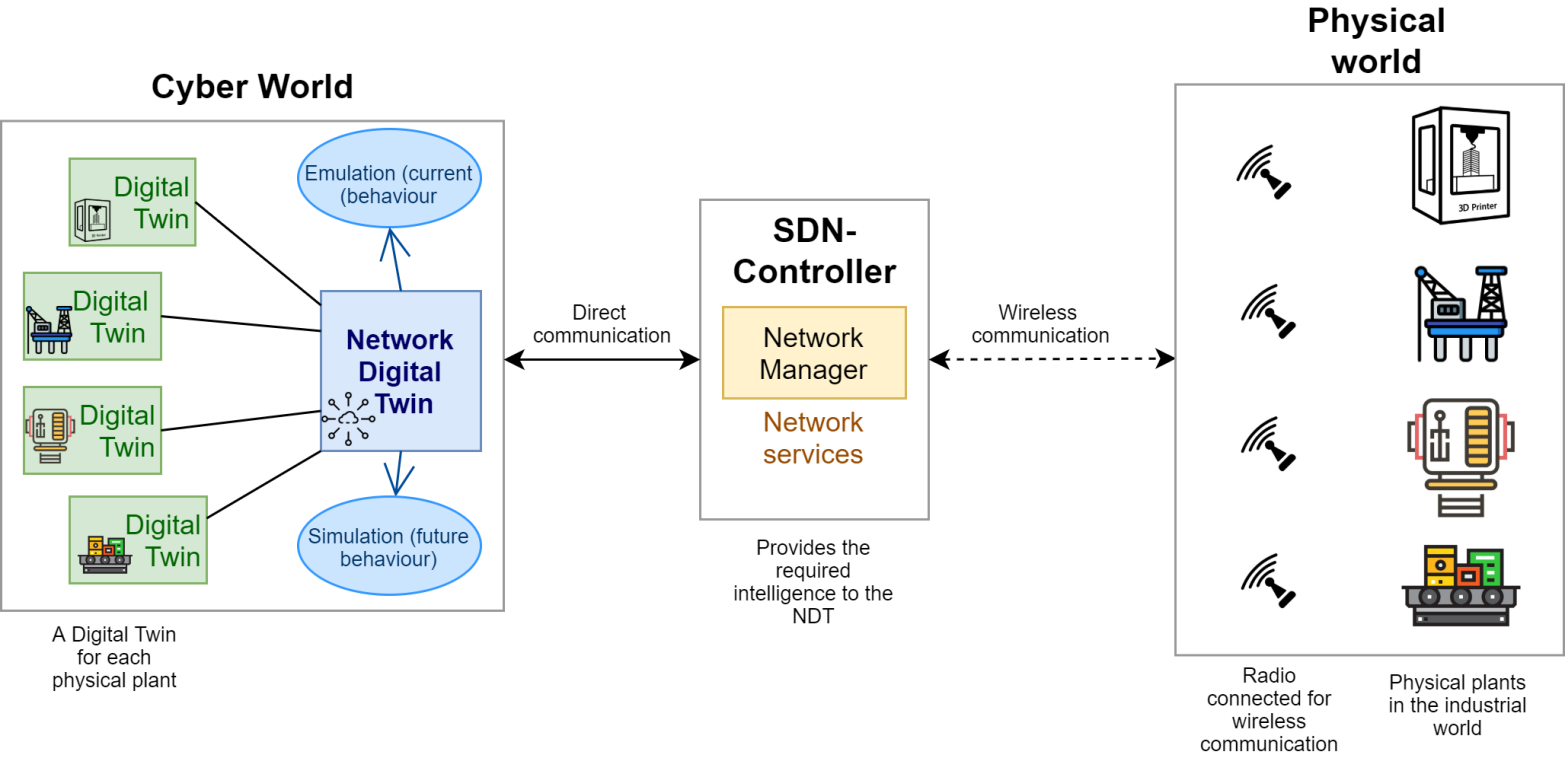
Digital Twin Networking based architecture for industry 4.0.

In order to implement the proposed design approach, we propose a holistic architecture composed of three main parts as shown in Figure 3. A physical world composed of physical plants which could be any industrial object/system such as a 3D printer, an oil platform, a conveyor, a machine, etc. Each physical plant is equipped with wireless sensor nodes that are responsible of collecting data on their operating conditions. The second part consists in the cyber world that integrates a digital twin for each industrial system present in the physical world all interacting with the NDT, the digital twin of the industrial network. The physical and cyber worlds are connected via an SDN controller that acts as a bridge between the two worlds, forwarding information flows from the cyber world to the physical world and vice-versa. The SDN paradigm is adopted since it facilitates the management of networks, enables network centralization, allows network programmability and also network slicing when combined with Network Function Virtualization (NFV). With this approach, all the data describing the physical network is captured by the SDN Controller that constructs the network topology model and provides the necessary intelligence to the NDT.

When it comes to practical implementation of our architecture, we need to consider the constrained nature of the IIoT in terms of computation and storage means as well as in terms of energy. By including an NDT in the architecture, more data has to be exchanged in a bidirectional way with more packets processing to ensure the synchronization between the physical and digital network. This leads to increasing the traffic load in the network and the nodes energy consumption. So, the challenge when implementing an NDT would be to find a trade-off between increasing energy consumption and ensuring the digital twinning operations. For example, the NDT would provoke more energy consumption in the early stages of the network operations (due to the high amount of information that should be exchanged to synchronize the two sides) but once it becomes stable, it can apply mechanisms that should increase the network’s remaining useful life.

In what follows, we give some of the benefits we can obtain when adopting this architecture in the design and the service phases. The latter includes production and maintenance.

### 3.1. Design Phase

With the proposed architecture, it is possible for the NDT to leverage collected data from the real world to provide insights on the changes to be made to the network solution being designed in order to ensure the intended operation and get the required performance, thus validating the solution more quickly. Moreover, data visualization and analysis tools can be implemented in the NDT which may help network designers to accurately interpret the behavior of a network protocol and analyze the interactive behaviors among the network components. More interestingly, the NDT would permit testing new network solutions under various conditions, having more accurate results than current simulation tools because the NDT can take into consideration the surrounding environmental conditions and provides more immersive experiments thanks to its permanent connection with the real world. This helps network designers to detect and eradicate the eventual surprising undesirable behaviors that could occur in the deployment environment. Last but not least, the NDT can interact continuously with industrial systems digital twins to get insights on the networking requirements of each one and adjust the network’s resource allocation policy according to that.

### 3.2. Service Phase

Our architecture allows a continuous evolution of the physical network. In fact, the NDT would provide continuous real-time remote network monitoring based on the information it receives from the physical network. In addition, predictive maintenance mechanisms could be implemented in the NDT using, for instance, AI algorithms and specific network policies could be applied to increase the network remaining useful life. Network diagnostics can also be ensured by the NDT. Based on the reports generated, it adapts continuously the implemented mechanism to improve their efficiency. Also, the NDT can take action to ensure that the requirements of network applications are answered by adapting the network configuration. For instance, when multiple network applications are running on the same network stack, the NDT can provide a network configuration that makes a balance between the current network capacity and the applications requirements.

## 4. Industrial Case Study

The proposed architecture can be applied in many DT-based architectures to allow an efficient connection between the real and the digital worlds. In the personalized production and distributed manufacturing context for instance, a digital twin for a connected micro smart factory is designed and implemented in [35]. The DT uses an IIoT network to ensure the synchronization with the physical manufacturing components. This synchronization allows the DT to monitor the present in real-time, to track the past, and make predictions to support decision-making for the future. The NDT concept can be included in this architecture to manage the IIoT network and boost its performance.

**Figure 4:**
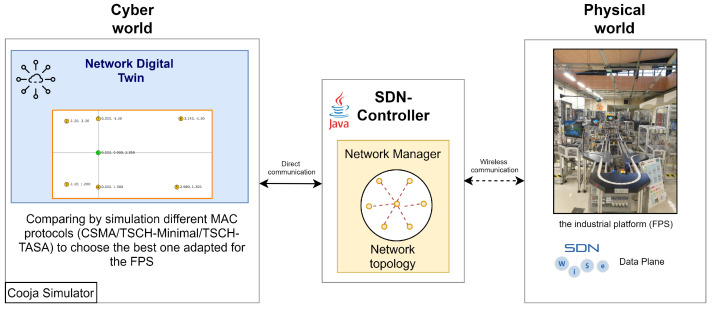
Practical architecture adapted to our industrial case study.

In order to validate the proposed Network Digital Twin architecture, we consider its application to the early design stage of an industrial project. The purpose of this project is to satisfy the real-time requirements of a control application that monitors the operation of a Flexible Production System (FPS) [36]. To do so, a WSN needs to be deployed to allow collecting information on the manufacturing operations carried out by the FPS. One raised question concerns which mechanism allows to meet our real-time requirements. As a preliminary step, a Network Digital Twin is built to assess the performance of the platform equipped with the WSN under three MAC protocols along with an oversampling mechanism. Figure 4 depicts the practical scheme of our proposed NDT architecture adapted to the considered industrial case study.

In the physical world, there is the industrial platform that consists in an FPS installed in approximately a 20 m^2^ area within our university. This platform aims to support teaching activities in automation engineering, industrial supervision, industrial communication, control and system integration. The FPS is composed of six assembly stations connected by a conveyor where each station is equipped with a Programmable Logic Controller (PLC). A sensor node is installed at each station in order to report information on its operation process to a central node suspended in the ceiling centrally above the FPS.

In order to carry out the design of our project, we proceed by creating an NDT to get insights on the most appropriate choices with regard to network protocols to satisfy the real-time constraints of our FPS. That is, in the cyber world, cooja simulator [37] is used in order to replicate the behavior of the WSN deployed in the physical world. To do so, the different distances between the sensor nodes and the central node (the Sink) are measured and the corresponding topology in cooja is reproduced as shown on the left side of Figure 4. Green node numbered 1 is the Sink and it is located at an altitude of 2.35 m with respect to sensor nodes.

In order to allow communication between the real and the cyber worlds, SDN-WISE [38,39] is used as it is more suitable to WSNs. SDN-WISE focuses on network flexibility and security with the goal of making WSNs modular in terms of communication and processing, reducing the amount of information exchanged between the sensor nodes and the SDN controller, and making the nodes programmable as finite state machines. The SDN-WISE controller keeps track of the network topology using a graph where vertices are the nodes and the edges are the links between these nodes. In SDN-WISE, the sink is the intermediary between the sensor nodes and the controller. It starts by broadcasting a packet called “beacon” that contains the identity of the sink that generated it, a battery level, and the current distance from the sink which is initially set to 0. A neighbor node, upon receiving such a packet, inserts the source node in its neighbor nodes list. If the current distance from the Sink is better than the one it holds then the source node becomes its next hop to the Sink. The current distance is incremented in the beacon packet before it is rebroadcast. After constructing its list of neighbors using the different “beacon” packets received, a node generates a “report” packet containing its current list of neighbors and sends it to the controller. This latter, uses report packets to construct a global view of the network. This protocol is run periodically to ensure that the controller always have an updated view of the network. The frequency of sending “beacon” and “report” packets are application specific and impacts the performance of the network.

The process of building a global view of the network is a key feature in the proposed architecture. Even if in the considered case study, the network topology was manually defined, one can make automatic topology discovery. This may be useful to consider already deployed networks in harsh environments. More interestingly, any change in the physical network, mainly during the service phase, would be detected by the controller which updates the digital replica consequently. These dynamics can be accommodated by the NDT and the running solution can be updated accordingly. In the proposed practical implementation, the network topology can be described within the XML (Extensible Markup Language) file that describes the scenario to run by the cooja simulator.

### 4.1. Real-Time Requirements

In an industrial environment, the control of automation applications is usually based on cyclic processes that run according to a predefined sampling. The sampling period must allow the control system to be updated while counting for the communication overhead. In order for the system to be updated every period, it must therefore be ensured that all communications arrive within the period. If a network message gets lost then the controller will be deprived of fresh input data and/or a remote control action will not be executed. Reliability and timeliness are of a paramount importance in an industrial application. Since, we aim to endow our FPS with a WSN, it is worth noting that real-time communication in WSNs is more challenging due to their severe constraints in terms of processing and communication means, in addition to the unreliable nature of the wireless medium and its shared access.

To overcome the above mentioned problem, a common solution is to apply an oversampling mechanism where a message is sent more than once in the sampling period to ensure the timely delivery of at least one copy of this message. The drawback of this solution is that sending multiple copies of each message within a given period would lead to network overloading due to congestion which decreases reliability and increases the experienced delays. As a result, a careful setting of the amount of redundancy to apply is crucial to ensure timeliness without affecting the application reliability.

Another solution consists in adopting a contention free access protocol. Recently, the IEEE 802.15.4e amendment [40] introduced several channel access modes that are contention free. This includes Time Slotted Channel Hopping (TSCH) which received the attention of both academia and industry due to its high performance in real-time constrained applications. TSCH targets application areas such as industrial automation and process control and offers support for multi-hop and multi-channel communications [41]. It has been designed to satisfy the requirements of IIoT applications as it provides time critical assurances and very high reliability [42]. It schedules data communication between network nodes by combining time slotted access with the channel hopping mechanism. The first mechanism avoids collisions between competing nodes, so it increases throughput and provides deterministic latency to applications. The channel hopping mechanism in turn allows multiple nodes to communicate simultaneously using different channels. Therefore, it increases the capacity and reliability of the network by mitigating the negative effects of interference.

In TSCH, nodes synchronize to a periodic slotframe composed of a number of timeslots, which is repeated throughout the network lifecycle. Communication between the nodes follows a schedule that is defined to allow the nodes to communicate as efficiently as possible. This schedule can be modeled by a matrix whose rows are the available channels and columns are the timeslots in a slotframe. Each cell of the matrix represents a specific link having as coordinates (ChannelOffset, SlotOffset) and can be reserved for a single link or shared by several links. The CSMA-CA algorithm is executed if a collision occurs in the latter case. The frequency in which two nodes can communicate in a timeslot is calculated as follows:

(1) $$f=F[\left(ASN+channelOffset\right)\%N_{channels}]$$

where ASN is the total number of timeslots that have elapsed since the start of network service, incremented in each timeslot. Function F can be implemented as a look-up table, usually it is defined as the hopping sequence specified in TSCH. For example, if four channels are used, the hopping sequence can be {15,20,25,26}. It should be mentioned that Equation (1) can return different frequencies for the same link in different timeslots, which ensures the channel hopping mechanism.

### 4.2. Scenarios and Metrics

To answer our question on which solution would satisfy the real-time requirements of our industrial platform, a progressive methodology is followed. Based on the obtained results at each step, we decide whether a new mechanism is to be investigated. CSMA and TSCH minimal schedule (The TSCH minimal schedule is composed of one slotframe with three timeslots and only the first one is used and shared between all nodes) are considered first as they are already provided in cooja. Afterwards, a centralized scheduling algorithm called TASA (Traffic Aware Scheduling Algorithm) [43] is implemented and evaluated at the SDN controller. Table 1 presents TASA parameters setting.

The platform’s refreshing time is 2 s so the main data rate is one packet every 2 s. To assess the oversampling mechanism, we considered both duplicating and triplicating a data packet within a 2-s observation period. The data transmission rate without oversampling is one packet every 2 s. In the case of duplication the rates becomes 1 pps (packet per second) where one packet is sent at the beginning of the period and the second message with an offset of one second. When triplication is performed, the first message is sent at the beginning of the period, the second with an offset of 2/3 s, and the third with a 4/3 s offset, resulting in a data rate of 3/2 pps. The purpose of these offsets is to avoid creating congestion in the output buffers. Thus in the case of the duplication for instance, the first message meets the requirements if it is received in less than two seconds, while the second message will only have one second to arrive within the time limit. It is sufficient for one of the messages to meet these constraints to conclude that the system has been refreshed. The reported data from the FPS to the controller are of boolean type, that is the payload of each transmitted packet is one byte long. Note that additionally ten bytes are used by the SDN-WISE header. Every scenario has been executed during 40 min and repeated four times with a different random seed in each run. The process of sending data packets starts after an initialization phase of six minutes.

In addition to two network performance indicators, namely packet delay and packet delivery ratio (PDR), an application performance indicator called the freshness indicator (FI) is considered. The packet delay is the difference between the time of its reception by the Sink and its transmission time by a sensor node. The PDR is the ratio of the number of received packets by the Sink to the number of sent packets by the different sensor nodes of the platform. The FI is defined as the ratio of the number of periods in which at least one packet is received by the Sink within the period duration to the total number of periods of the whole simulation. In what follows, the simulations results are presented using box plots in order to visualize the distribution of obtained data points. Mean values are also plotted as empty squares inside the box plots.

### 4.3. Simulation Results

#### 4.3.1. CSMA

**Figure 5:**
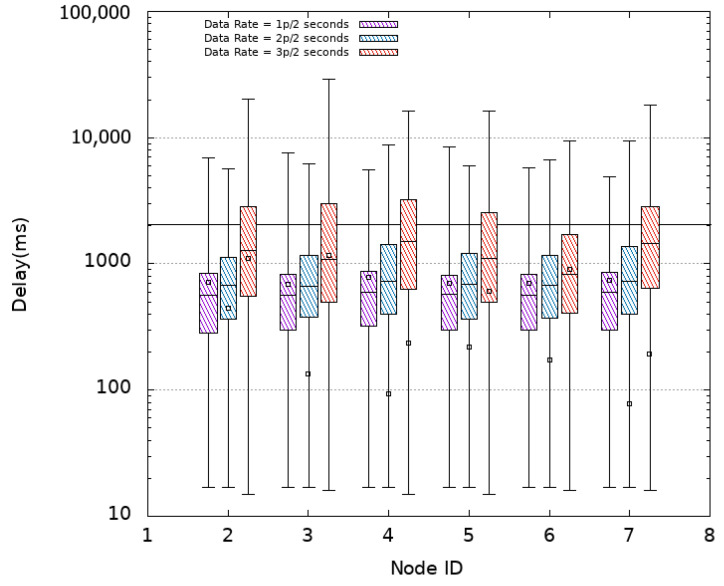
Packet Delay—CSMA.

**Figure 6:**
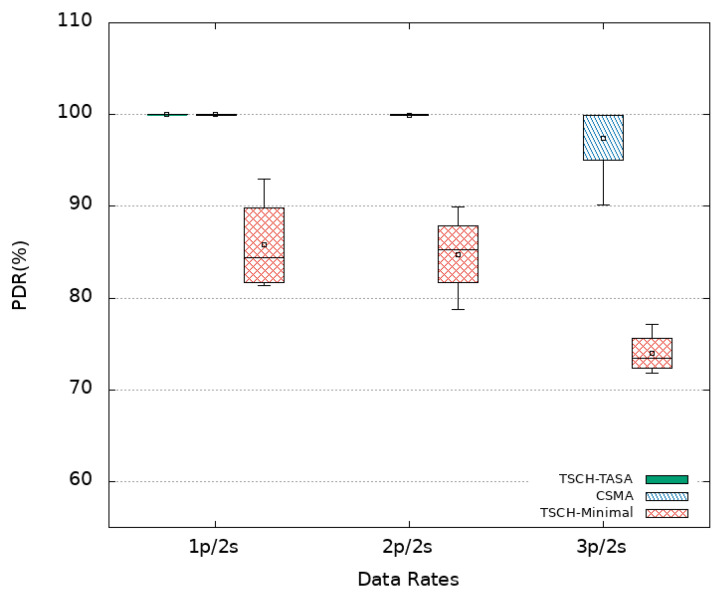
PDR results.

**Figure 7:**
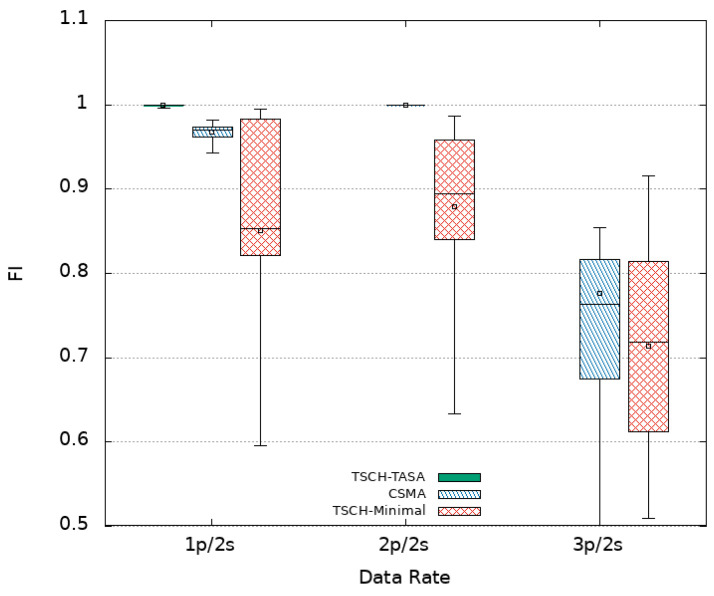
FI results.

Experiments are first conducted using CSMA as MAC protocol without oversampling (i.e., using a data rate of 1p/2s) then an oversampling with 2p/2s and 3p/2s data rates is considered. Figure 5 plots the obtained delays (log-scale) when using CSMA for each sensor node numbered 2 to 7 in the *x*-axis. The horizontal line at ordinate 2 s recalls the refreshing time period. It is noted that all nodes obtain similar latencies for the different settings. This is due to the fact that they are located almost at similar distances from the Sink reached in one hop resulting in a star topology. In the absence of oversampling (1p/2s), an average delay of 719 ms with an average median delay of 575 ms is obtained. However, it is observed that some packets (about 6 % as shown in Table 2) arrive after a delay that exceeds 2 s. This translates into an average freshness indicator of 97% while the PDR achieves its maximum value (100%). The distribution of PDR and FI obtained in the different experimented MAC protocols with or without oversampling are presented in Figure 6 and Figure 7, respectively.

Since no loss is experienced, the duplication of the transmission rate (to 2p/2s) can be afforded by sending each packet twice in the 2-s period window. Not only a PDR of 100% is kept but also a 100% of freshness is achieved for all nodes. Delay results show an increase of the mean values, with an average of 931 ms, but the median values decreases to 696 ms. This latter explains the obtained results of the FI even if some packets still arrive out of time as depicted in Figure 5. Certainly, at least one copy of each message is received and losses only concern duplicates. Duplication increases the probability that a message is received within the 2-s window. Given the obtained results, triplicating seem to be of no interest. This is confirmed by our experiments where an increase is noticed in the delay results with 33% of packet delays exceed 2 s and a decrease in terms of PDR as an average value of 97.5% is obtained. Moreover, a significant decrease in the FI (77% in average) is to be noted. The increase of delay values is due to more experienced collisions that result from overloading the network by higher data rates. In fact, CSMA uses a contention window that imposes a waiting time (back_off time) for nodes to avoid collisions. This window doubles in size every time a collision occurs which causes greater delay values.

#### 4.3.2. Minimal TSCH

**Figure 8:**
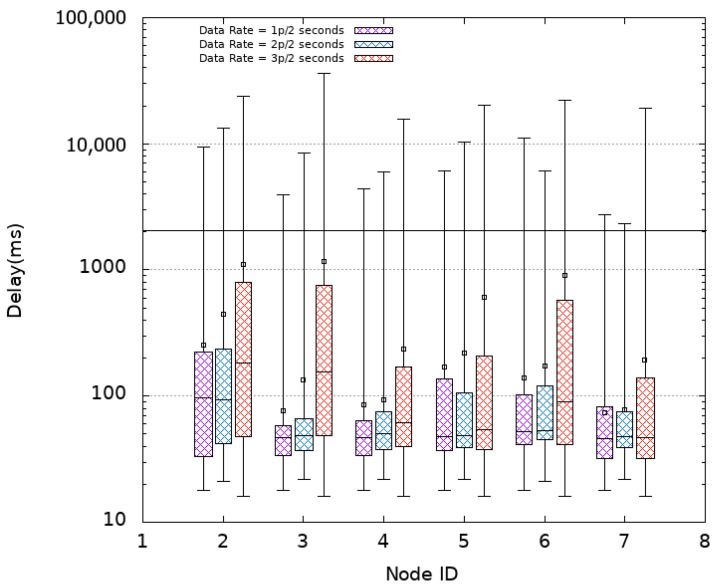
Packet Delay—Minimal TSCH.

As opposed to CSMA, TSCH has been introduced to meet the real-time needs of industrial applications as the nodes do not compete to access the medium. We decided to consider a TSCH-based MAC protocol in our study. Its minimal scheduling available in the Cooja simulator is considered first. Figure 8 presents the obtained results where lower delays can be observed when compared to CSMA. For instance, without oversampling (1p/2s), it is obtain in average, a mean and median delay of 133 ms and 56 ms, respectively. Among received packets, only 1% are out of time as shown in Table 2. Despite that, the achieved PDR and FI as shown in Figure 6 and Figure 7 are lower with an average value of 86% and 85%, respectively. Even worse, the minimum value for FI may drop as low as 60% because of 1% of the packets that arrives with a delay that exceeds 2 s.

At this stage, is it worth applying oversampling? A priori no, but we further increased the transmission rate to confirm our assumptions. When duplication (2p/2s) is considered, the delays are slightly increased when compared to the case of 1p/2s with an average of 189 ms and a median of 57 ms. Duplication allows a slight improvement of the FI from 85% to 87% of the periods getting fresh data as shown in Figure 7 but decreases the PDR to 85% as shown in Figure 6. It is noted that the FI box plot spreads over a wider range with a minimum value of 66% and a maximum value of 98%. The former value results from the 2% of packets with delay exceeding 2 s and the latter (as well as the median) value is due to the fact that the probability of receiving a data packet within the two-second window is increased. Going further and triplicating messages (3p/2s) is not worth doing either since higher delays are experienced with lower PDR and FI values as we obtain 74% and 71% in average, respectively.

The increase in delay when applying oversampling is due to the fact that each sensor node has more packets to transmit within the same duration which adds to the delay, the time each node spends waiting turn to transmit. The experienced losses can be explained by the fact that the scheduling with Minimal TSCH is not optimal.

#### 4.3.3. TSCH TASA

**Figure 9:**
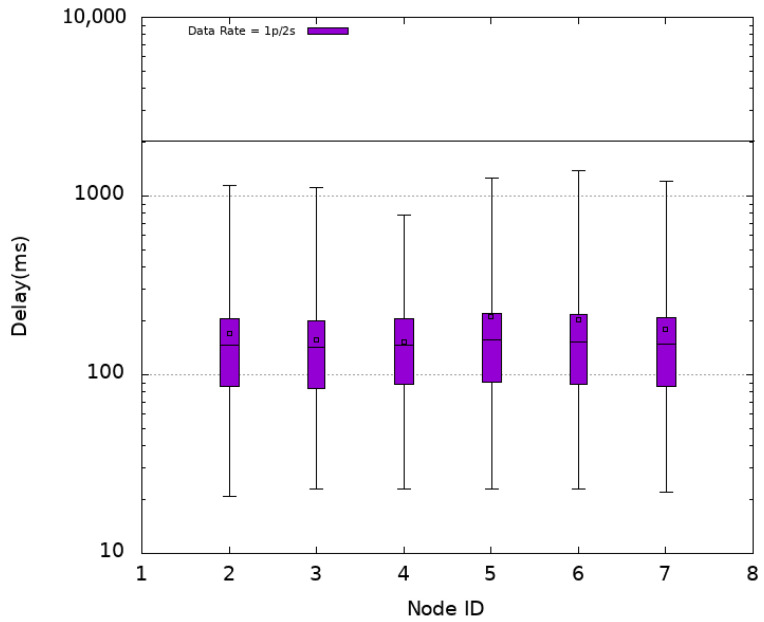
TSCH/TASA delay results.

The obtained results when using TSCH with its minimal schedule is inefficient and can not suit our needs. This is why, we considered implementing and testing a more advanced scheduling algorithm called TASA. As done with the previously considered MAC protocols, we began by experimenting TASA without oversampling i.e., using a data rate of 1p/2s. The obtained delays as shown in Figure 9 fully satisfy our real time requirement as all packets arrive with a delay that does not exceed 2 s. Even better, almost 75% of the packets experience a latency below the 200 ms. An overall average and a median of 178 ms and 148 ms are recorded. Both the PDR and the FI achieve their maximum values (100%) as shown in Figure 6 and Figure 7, respectively. As a result, this configuration (i.e., TASA without oversampling) answers perfectly the real-time requirements of the industrial system of interest, the object of this study. Both reliability and timeliness are achieved with the minimum transmission rate. This allows to not consume extra energy that could have been consumed by the oversampling for packets replications that are no longer needed.

Following the obtained results, the corresponding firmware is uploaded to the FPS sensors using the SDN controller. This latter will have in charge to monitor their operation based on the reports sent periodically. When an anomaly is detected, the designer is alerted to correct and/or adapt the current implementation. The newly obtained firmware is then uploaded and a new “agile” iteration is undertaken. It is worth noting that AI algorithms can be leveraged to automate this process operations as much as possible in order to gain more efficiency.

## 5. Conclusions

In this paper, we proposed a holistic digital twin architecture for the IIoT where the network component is considered through the adoption of the concept of a Network Digital Twin (NDT). The aim is to enable quick validation of networking solutions in an industrial environment since the NDT is continuously linked to the physical world from the early design stage to the production phase. Moreover, the NDT provides a continuous evolution of the industrial network by exploiting the data collected along with AI algorithms to enhance the networking performance, prevent network failure and increase the network remaining useful life. The proposed NDT can be included in any DT-based architecture where a communication network is required in the physical world.

We validated the proposed architecture through its application to the early design stage of an industrial project with real-time requirements. Precisely, we used the concept of NDT to assess different scenarios in order to choose the best suited communication mechanism satisfying the real time requirements of the FPS application. We mainly found that oversampling is no longer required when using a TDMA-like MAC protocol such as TASA. The next step consists in undertaking subsequent iterations to consider the effectiveness of the proposed solution in the real network. More elaborated network topologies will be considered to assess the scalability of the proposed solution to more complex industrial systems. Moreover, the adaptive character of the NDT in response to failures in the network in addition to its dynamics will be investigated possibly using AI-based solutions for network diagnosis and prediction. A new communication scheduling algorithm will be designed to further improve the performance of the industrial application. This would include an adaptive TSCH scheduler that exploits the data collected as an input to the NDT to provide the most efficient communication scheduling to the sensor nodes. Finally, it is envisaged to enhance the proposed architecture where a tight collaboration between the different DTs including the NDT with the aim of achieving a better orchestration of the whole industrial system.

## Figures and Tables

**Table 1:** Parameters used for TSCH with the Traffic Aware Scheduling Algorithm (TASA) scenario.

Parameter	Value
Period of sending “*beacon*” packets	5 s
Period of sending “*report*” packets	10 s
Slotframe size	15 timeslots
Timeslot’s duration	10 ms

**Table 2:** Percentages of packet delays exceeding 2 s for each scenario along with their respective PDR values.

	Data Rate	1p/2s		2p/2s		3p/2s	
Protocol							
percentage of		>2 s	PDR	>2 s	PDR	>2 s	PDR
CSMA		6%	100%	11%	100%	33%	97.5%
TSCH minimal		1%	86%	2%	85%	9%	74%

## Data Availability

The data presented in this study are available on request from the corresponding author.
